# Management of snake bite during third trimester of pregnancy with coagulopathy and delivery of a live baby in resource-limited setting in Nepal: a case report

**DOI:** 10.1093/omcr/omac105

**Published:** 2022-10-22

**Authors:** Ashwini Gupta, Sudeep Bhandari, Ayush Anand, Sanjib Kumar Sharma, Arun Gautam, K C Priyanka, Neeraj Acharya, Sweta Singh

**Affiliations:** B. P. Koirala Institute of Health Sciences, Dharan, Nepal; Department of Internal Medicine, B. P. Koirala Institute of Health Sciences, Dharan, Nepal; B. P. Koirala Institute of Health Sciences, Dharan, Nepal; Department of Internal Medicine, B. P. Koirala Institute of Health Sciences, Dharan, Nepal; Department of Internal Medicine, B. P. Koirala Institute of Health Sciences, Dharan, Nepal; Department of Internal Medicine, B. P. Koirala Institute of Health Sciences, Dharan, Nepal; Department of Internal Medicine, B. P. Koirala Institute of Health Sciences, Dharan, Nepal; B. P. Koirala Institute of Health Sciences, Dharan, Nepal

## Abstract

We reported a case of snakebite in an 18-year-old woman, Gravida 2 Para 1+0 in the third trimester of pregnancy who presented with pain and swelling over the left hand and forearm and vaginal spotting. The laboratory investigations revealed coagulopathy attributed to green pit viper envenomation. On the fourth day of admission, the patient developed sudden abdominal pain and massive per vaginal bleeding with haemorrhagic shock, most likely abruptio placentae. In Nepal, no anti-snake venom has been developed for green pit-viper. So, she was managed conservatively, including blood transfusion, and delivered a single live female baby without any foetal complications. The patient was discharged along with the baby after 8 days of hospitalization. This case demonstrated that vigilant observation and appropriate resuscitation with fluids and blood products could save mother and baby in pit viper envenomation cases in settings where specific anti-snake venom is unavailable.

## INTRODUCTION

Snakebite, with an estimated incidence of 251 per 100 000 snakebites yearly, is a serious public health concern in Nepal [1]. Green pit vipers and mountain pit vipers are commonly found poisonous snake species in Nepal’s hills and highlands. [2]. The constituents of viper snake venom can lead to impaired coagulation profile and eventually cause consumption coagulopathy [3]. A review by Langley *et al*. revealed that snake bites in pregnancy could lead to various maternal and foetal complications such as teratogenesis, spontaneous miscarriage, abruptio placenta, preterm labour and intrauterine foetal death [4]. Though the reports of snakebite envenomation in pregnancy ranged from 0.4 to 1.8% of snake bite cases [5], the overall maternal mortality and foetal death rates were ~4 and 20%, respectively [4]. For the management of snake bites, polyvalent anti-snake venom is imported from India, which is effective only against envenomation by common krait, cobra, Russell’s viper and saw-scaled viper [6]. Moreover, the use of anti-snake venom is not without complications. A study by Sharma *et al*. reported anaphylaxis in 8.4% of patients and death in 5.6% of patients attributed to snake antivenom administration [7]. Hence, vigilant observation and supportive management remain the mainstay of management in resource-limited settings where specific anti-snake venom is unavailable. Here, we describe the successful management of a patient with coagulopathy attributed to envenomation by pit viper in the third trimester of pregnancy.

## CASE REPORT

An 18-year-old female (Gravida 2 Parity 1 Neonatal Death 1 and Living 0) at 33 weeks 3 days period of gestation presented to Obstetrics Emergency 18 h following a snake bite; with complaints of pain and swelling over the left hand and forearm along with per vaginal spotting since last 7 h.

**Figure 1:**
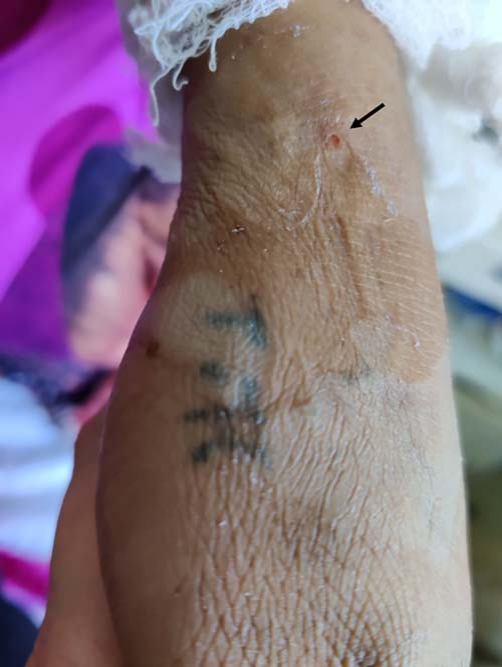
Fang mark on the flexor aspect of the left forearm.

She was hemodynamically stable and perceived adequate foetal movements. There were three fang marks on the flexor aspect of the left forearm (Fig. 1). She did not have any spontaneous bleeding manifestations, including that from the bite mark. The external os seemed closed on per-speculum examination with no active bleeding. There were no signs of neurotoxic envenomation. No history of any chronic illness or bleeding diathesis in the patient. Her initial laboratory investigations (Table 1) revealed anaemia, leukocytosis and impaired coagulation profile (Table 1). She was managed conservatively by intravenous normal saline and shifted to the medicine ward.

**Table 1:** Laboratory investigations of the patient during the hospital stay

Investigations	06\11	06\14	06\14	06\15	06\16	06\17	06\18
CBC (cells/mm^3^)	13 800	15 500	18 800	8600	8400	6900	8400
DLC (%)	N70 L29	N86 L10	N87 L7	N84 L08	N85 L05	N74 L15	N67 L20
Platelet (cells/mm^3^)	161 000	95 000	44 000	50 000	95 000	142 000	191 000
Hb (g/dl)	10.7	8.1	5.7	5.8	6.9	6.5	9.7
PCV (%)	33.5	25.8	18.1	18.1	19.8	20	29.7
PTT (s)	60	48	16	18			
INR	> 4.5	3.6	1.12	1			
WBCT20 (min)	>40						

CBC = complete blood cell count; DLC = differential leucocyte count; N = percentage of neutrophil; L = percentage of lymphocyte; Hb = haemoglobin; PCV = packed cell volume; PTT = prothrombin time; INR = International normalized ratio; WBCT = 20-min whole blood clotting test.

The first 3 days of her stay in the medicine ward were uneventful, and she perceived adequate foetal movements. As advised by the obstetrician, she was given dexamethasone for foetal lung maturation and various oral and intravenous drugs (Table 2). On the fourth day, she suddenly developed pain abdomen and massive vaginal bleeding with haemorrhagic shock. She was resuscitated with intravenous fluids, blood products and anticoagulants (Table 2). Based on the clinical evaluation by obstetrician, she was suspected of having abruptio placenta and was immediately shifted to the maternal intensive care unit (MICU) for further management.

**Table 2:** Treatment of the patient during the hospital stay

Treatment during first 3 days of stay in Medicine Ward • Dexamethasone 6 mg intravenously 4 doses with 12 hbetween each dose for foetal lung maturation • Flucloxacillin 500 mg orally QID • Ferrous ascorbate 100 mg orally and folic acid 1.5 mg orally • Ranitidine 150 mg OD intravenously
Treatment on the fourth day of hospital stay before the shift to MICU • Intravenous injections of Vitamin K 10 mg stat and Tranexamic acid 1 gm stat • 12 pints of 0.9% normal saline • 1 pint of whole blood • 4 pints of packed cells • 4 pints of fresh frozen plasma

In MICU, she was planned for termination of pregnancy, and induction was done with artificial rupture of membrane. The delivery was successful with delivery of a live baby. During the hospital stay, she received two more pints of whole blood. Also, she developed post-partum fever for 2 days. Chest-X ray, urine culture, blood culture and transvaginal ultrasonography were carried out to investigate the cause of post-partum fever. However, the results did not suggest of any infectious or non-infectious causes. Hence, the post-partum fever was attributed to thrombophlebitis, which improved with dressing and heparin ointment. By the fifth day of her hospital stay, her coagulation profile and complete blood cell count were normal (Table 1). The patient was prescribed a combination of Capsule Ferrous Ascorbate 100 mg plus Folic Acid 1.5 mg for 6 weeks for correction of anaemia and was discharged along with her baby.

## DISCUSSION

Green pit viper envenomation is common in Nepal, and the lack of specific anti venom [2] makes it challenging to manage patients with coagulopathy. In addition, envenomation during pregnancy is associated with adverse maternal and foetal outcomes [4]. The coagulating agent in the toxin can reach the decidua-placental cleavage zone and may start a dissociation leading to placental abruption [8]. Similarly, placental abruption was suspected in our case as the patients developed abdominal pain and heavy vaginal bleeding. Also, excessive bleeding in patients can result in loss of anticoagulants and procoagulants, leading to an increased risk of consumption coagulopathy [9]. And, haemodilution due to intravenous fluids can worsen the condition even further [9]. A review by Bollinger *et al*. revealed that consumption coagulopathy could be prevented by transfusion of packed RBCs along with fresh frozen plasma, cryoprecipitate, fibrinogen concentrate and factor XIII concentrate [9]. Furthermore, in low-resource settings where blood products are not easily procurable, fresh whole blood is transfused as an alternative to clotting factors, particularly in patients with or at risk of anaemia [3]. In our case, the patient was resuscitated with fluids, blood products and anticoagulants, followed by timely labour induction. We used fresh whole blood and fresh frozen plasma along with packed red blood cells for a successful resuscitation without consumption coagulopathy. Our case highlighted the importance of early intervention, symptomatic management and vigilance required to manage snakebite in pregnancy, particularly in low-resource settings where specific anti-snake venom is not available.

## CONCLUSION

This case reported a successful outcome in managing the patient with coagulopathy following a snake bite during the third trimester of pregnancy. The mainstay of management was supportive treatment and vigilance along with timely fluids resuscitation, administration of steroids for foetal maturity, induction of labour and cautious use of blood products to save both mother and baby. We recommend that the symptomatic approach and vigilance be practised in managing snake bites in pregnancy in settings where specific anti-snake venom is unavailable. Also, stakeholders should make efforts to train the healthcare workers accordingly and make efforts for the development of specific anti-snake venom.

## CONFLICT OF INTEREST STATEMENT

The authors have no conflicts of interest to declare.

## FUNDING

The authors did not receive any funding for this paper.

## ETHICAL APPROVAL

Ethical approval was not required for this paper.

## CONSENT

A written informed consent was obtained from the patient for the publication of this case report based on the journal’s policy.

## GUARANTOR

The Guarantor of the paper is Prof. Dr Sanjib Kumar Sharma.
